# Gene Regulatory Scenarios of Primary 1,25-Dihydroxyvitamin D_3_ Target Genes in a Human Myeloid Leukemia Cell Line

**DOI:** 10.3390/cancers5041221

**Published:** 2013-10-16

**Authors:** Jussi Ryynänen, Sabine Seuter, Moray J. Campbell, Carsten Carlberg

**Affiliations:** 1 School of Medicine, Institute of Biomedicine, University of Eastern Finland, POB 1627, Kuopio FI-70211, Finland; E-Mails: jussi.ryynanen@uef.fi (J.R.), sabine.seuter@uef.fi (S.S.); 2 Department of Pharmacology and Therapeutics, Roswell Park Cancer Institute, Elm and Carlton Streets, Buffalo, NY 14263, USA; E-Mail: Moray.Campbell@RoswellPark.org

**Keywords:** vitamin D receptor, vitamin D, *G0S2*, *CDKN1A*, *MYC*, genomics, chromatin, gene regulation

## Abstract

Genome- and transcriptome-wide data has significantly increased the amount of available information about primary 1,25-dihydroxyvitamin D_3_ (1,25(OH)_2_D_3_) target genes in cancer cell models, such as human THP-1 myelomonocytic leukemia cells. In this study, we investigated the genes *G0S2*, *CDKN1A* and *MYC* as master examples of primary vitamin D receptor (VDR) targets being involved in the control of cellular proliferation. The chromosomal domains of *G0S2* and *CDKN1A* are 140–170 kb in size and contain one and three VDR binding sites, respectively. This is rather compact compared to the *MYC* locus that is 15 times larger and accommodates four VDR binding sites. All eight VDR binding sites were studied by chromatin immunoprecipitation in THP-1 cells. Interestingly, the site closest to the transcription start site of the down-regulated *MYC* gene showed 1,25(OH)_2_D_3_-dependent reduction of VDR binding and is not associated with open chromatin. Four of the other seven VDR binding regions contain a typical DR3-type VDR binding sequence, three of which are also occupied with VDR in macrophage-like cells. In conclusion, the three examples suggest that each VDR target gene has an individual regulatory scenario. However, some general components of these scenarios may be useful for the development of new therapy regimens.

## 1. Introduction

The hormonal form of vitamin D_3_, 1α,25-dihydroxyvitamin D_3_ (1,25(OH)_2_D_3_), has the interesting property of directly activating one defined transcription factor, the vitamin D receptor (VDR) [1]. In common with other well-understood and important transcription factors, information on the genome-wide binding profile of VDR was consequently highly desired. As a result, the method of chromatin immunoprecipitation (ChIP) coupled with massive parallel sequencing (ChIP-seq) [2]) was applied in several human cellular models. Three years ago, VDR ChIP-seq was first reported for the immortalized B lymphocyte cell lines GM10855 and GM10861 (obtained from Caucasian female individuals of the HapMap project) [3], then for the monocytic cell line THP-1 (derived from a male infant with acute myelomonocytic leukemia) [4], later for the colorectal adenocarcinoma cell line LS180 (obtained from a Caucasian female) [5] and finally for the spontaneously immortalized hepatic stellate cell line LX2 (purified from normal human liver) [6]. In addition, very recently there was the first report of genome-wide VDR binding in primary CD4^+^ T-lymphocytes (obtained from nine healthy human volunteers) [7]. The number of statistically significant VDR binding sites of the respective datasets varied between a few hundreds and more than 10,000, but only a low percentage of them are identical, when comparing two or more cellular models [8]. These models aim to cover the spectrum of responses to 1,25(OH)_2_D_3_ observed in health and disease. The immortalized cell models, lymphoblastoid and LX2 cells, essentially capture normal VDR signaling, while THP-1 leukemia cells display significant phenotypic responses towards 1,25(OH)_2_D_3_ exposure in terms of triggering differentiation. In contrast, LS180 cells have a resistant phenotype with loss of sensitivity towards the anti-proliferative actions of 1,25(OH)_2_D_3_, *i.e.*, they are less suited as a representative cancer model.

Traditionally within the field of VDR research, single gene studies had supported the concept that the VDR binds preferentially to sequences formed by a direct repeat of two hexameric binding motifs spaced by three nucleotides (DR3) [9,10]; this binding motif echoed the arrangement for other nuclear receptors. However, the agnostic analyses of genome-wide VDR binding reveals that DR3-type sequences with a high similarity score were identified at the summits of only 30% of all VDR ChIP-seq peaks [8]. This suggests that there must be alternative mechanisms by which the VDR contacts its genomic targets than forming heterodimers with the retinoid X receptor on DR3-type sequences. This may involve heterodimerization with other transcription factors on different types of sequence or may even be independent of direct binding of the receptor to DNA [11]. The latter mechanism was first demonstrated for mutated p53, which can bind VDR [12].

In order to obtain access to its genomic binding sites, VDR has first to overcome the intrinsic repressive nature of chromatin [13,14]. At a lower rate, the VDR is able to contact genomic DNA already in the absence of 1,25(OH)_2_D_3_ and then preferentially forms complexes with co-repressor proteins [15,16] and chromatin modifying enzymes, such as histone deactylases (HDACs) [17,18]. However, the binding of 1,25(OH)_2_D_3_ to VDR’s ligand-binding domain induces a conformational change to the latter, so that VDR changes its interaction partners, a few of which have chromatin modifying activity like histone acetylation [19]. Therefore, the interaction of VDR with chromatin and its modifying enzymes is a central element in 1,25(OH)_2_D_3_ signaling [20]. HDAC inhibitors, some of which are already in clinical application [21], have the potential to interfere with the actions of VDR on chromatin. Accordingly, primary 1,25(OH)_2_D_3_ target genes had been distinguished into those that are up-regulated, down-regulated or not affected by HDAC inhibitor treatment [22].

When a transcriptional start site (TSS) lies within open chromatin, a basal transcriptional machinery complex is able to assemble there. In cases when ligand-activated VDR binds to the same chromosomal domain, the looping of the receptor towards the core promoter region can initiate or enhance the transcription of the respective gene. The method Formaldehyde-Assisted Isolation of Regulatory Elements (FAIRE) combined with massive parallel sequencing (FAIRE-seq) allows a genome-wide detection of these open chromatin regions [23,24]. Therefore, combined with VDR ChIP-seq data this method allows the characterization of the genomic regions of primary VDR target genes.

For this study, we have selected THP-1 cells as an attractive leukemia model, as it is the only one for which genome-wide VDR data are available. We present analyses of the three primary 1,25(OH)_2_D_3_ target genes G_0_/G_1_ switch 2 (*G0S2*), cyclin-dependent kinase inhibitor 1A (*CDKN1A*) and v-myc avian myelocytomatosis viral oncogene homolog (*MYC*) in terms of architecture of their TSS and a number of VDR binding sites. Involving genome-wide data in the characterization of 1,25(OH)_2_D_3_ target genes participating in the control of cellular growth may help to identify the most important components in cancer-related 1,25(OH)_2_D_3_ signaling.

## 2. Experimental

### 2.1. Cell Culture

The human monocytic cell line THP-1 [25] was grown in RPMI 1640 medium supplemented with 10% fetal calf serum, 2 mM L-glutamine, 0.1 mg/mL streptomycin and 100 U/mL penicillin and the cells were kept at 37 °C in a humidified 95% air/5% CO_2_ incubator. Prior to mRNA or chromatin extraction the cells were grown overnight in phenol red-free RPMI 1640 medium supplemented with 5% charcoal-stripped fetal calf serum. After 72 h treatment with 20 nM phorbol 12-myristate 13-acetate (PMA, Sigma-Aldrich, Helsinki, Finland) THP-1 cells become adherent and differentiate into a mature macrophage-like phenotype [26]. In 1,25(OH)_2_D_3_ stimulation experiments, cells were treated with 10 nM 1,25(OH)_2_D_3_ (Sigma-Aldrich) or solvent (0.001% EtOH). For HDAC inhibition experiments, cells were stimulated with 300 nM trichostatin A (TsA), 2 µM suberoylanilide hydroxamic acid (SAHA), 1 mM valproic acid (VPA, all compounds from Sigma-Aldrich), 100 nM 1,25(OH)_2_D_3_ or solvent (0.16% EtOH or 0.02% DMSO).

### 2.2. RNA Extraction, cDNA Synthesis and qPCR

Total RNA was extracted using the High Pure RNA Isolation Kit (Roche, Espoo, Finland) or the Quick RNA Miniprep Kit (Zymo Research, Espoo, Finland). For cDNA synthesis the Transcriptor First Strand cDNA Synthesis Kit (Roche) was applied, where total RNA and oligo(dT)18 primers were denaturated at 65 °C and reverse transcription was carried out for 30 min at 55 °C. qPCR reactions were performed using 250 nM of reverse and forward primers and the LightCycler 480 SYBRGreen I Master mix (Roche). The hotstart Taq polymerase was activated for 10 min at 95 °C, followed by 43 amplification cycles of 20 s denaturation at 95 °C, 15 s annealing at primer-specific temperatures (Supplementary Table S1) and 15 s elongation at 72 °C and a final elongation for 10 min at 72 °C. PCR product specificity was monitored using post-PCR melt curve analysis. Relative expression levels of the target genes were determined using the formula 2^−Ct^ and were normalized to the internal reference genes *B2M*, *GAPDH* and *HPRT1* as determined by the geNorm algorithm [27]. Briefly, the arithmetic mean of replicated Ct values for each reference gene was transformed to a relative quantity (Q) with the formula Q = 2^ΔCt^ = 2^(calibratorCt − sampleCt)^ by using the sample with the highest expression as a calibrator. For normalization, the relative expression level was divided by the normalization factor that is the geometric mean of the relative quantities (Q) of the three reference genes.

### 2.3. ChIP

After treatment of cells, nuclear proteins were cross-linked to DNA by adding formaldehyde directly to the medium to a final concentration of 1% and incubating for 8 min at room temperature on a rocking platform. Cross-linking was stopped by adding glycine to a final concentration of 0.125 M and incubating for 5 min at room temperature on a rocking platform. The cells were collected, washed with ice-cold PBS and resuspended in lysis buffer (1% SDS, 10 mM EDTA, protease inhibitors, 50 mM Tris-HCl, pH 8.1) and the lysates were sonicated with a Bioruptor Plus (Diagenode, Liege, Belgium) to result in DNA fragments of 200 to 400 bp. Cellular debris was removed by centrifugation. For output samples, aliquots of the lysate were diluted in ChIP dilution buffer (0.01% SDS, 1.1% Triton X-100, 1.2 mM EDTA, 167 mM NaCl, protease inhibitors, 250 µg/mL BSA, 16.7 mM Tris-HCl, pH 8.1). For input samples, the lysate was diluted 1:10 in ChIP dilution buffer without protease inhibitors and BSA. Anti-VDR antibody (sc-1008X, Santa Cruz Biotechnology, Heidelberg, Germany) or non-specific IgG (12-370, Millipore, Espoo, Finland) were bound for 3 h to Magna ChIP™ Protein A Magnetic Beads (Millipore). The pre-formed bead-antibody complexes were then washed with ChIP dilution buffer and added to the output chromatin aliquots. The samples were incubated overnight at 4 °C on a rotating platform to form and collect the immuno-complexes. The beads were washed sequentially for 4 min with the following buffers: low salt wash buffer (0.1% SDS, 1% Triton X-100, 2 mM EDTA, 150 mM NaCl, 20 mM Tris-HCl, pH 8.1), high salt wash buffer (0.1% SDS, 1% Triton X-100, 2 mM EDTA, 500 mM NaCl, 20 mM Tris-HCl, pH 8.1) and LiCl wash buffer (0.25 M LiCl, 1% Nonidet P-40, 1% sodium deoxycholate, 1 mM EDTA, 10 mM Tris-HCl, pH 8.1). Finally, the beads were washed twice with TE buffer (1 mM EDTA, 10 mM Tris-HCl, pH 8.0) and the immune complexes were eluted twice using elution buffer (1% SDS, 100 mM NaHCO_3_) for 20 min at room temperature with rotation. Both output and input samples were reverse cross-linked for 5 h at 65 °C in the presence of proteinase K (Roche). The DNA was isolated with the ChIP DNA Clean & Concentrator Kit (Zymo Research). Selected genomic regions containing VDR peaks were analyzed by qPCR using equal DNA amounts of chromatin fragments, 250 nM of reverse and forward primers and the LightCycler 480 SYBRGreen I master mix. The qPCR reactions were performed using the following profile: 10 min at 95 °C, followed by 43 cycles of 20 s at 95 °C, 15 s annealing at primer-specific temperatures (Supplementary Table S2) and 15 s at 72 °C, and a final amplification step of 10 min at 72 °C. The results were related to input by using the formula E^−(ΔCt)^ *100, where E = amplification efficiency and ΔCt = Ct_(output)_ − Ct_(input)_.

## 3. Results

### 3.1. Transcription of G0S2, CDKN1A and MYC in Monocyte- and Macrophage-Like Cells

Our microarray and ChIP-seq datasets from undifferentiated THP-1 cells (monocyte-like cells) [7] list 638 primary 1,25(OH)_2_D_3_ target genes and 2,340 genomic VDR binding sites. We screened these lists for well-characterized genes being involved in the control of cellular proliferation and selected *G0S2* and *CDKN1A*, because they were significantly up-regulated by 1,25(OH)_2_D_3_ and displayed one and three VDR peaks, respectively, in reasonable vicinity to their TSS region. For the *MYC* gene we identified even four VDR peaks, although our microarray data did not indicate any significant regulation of the gene. However, we knew from our previous study [28] in RWPE-1 immortalized prostate cells that *MYC* is a primary down-regulated gene.

**Figure S1:**
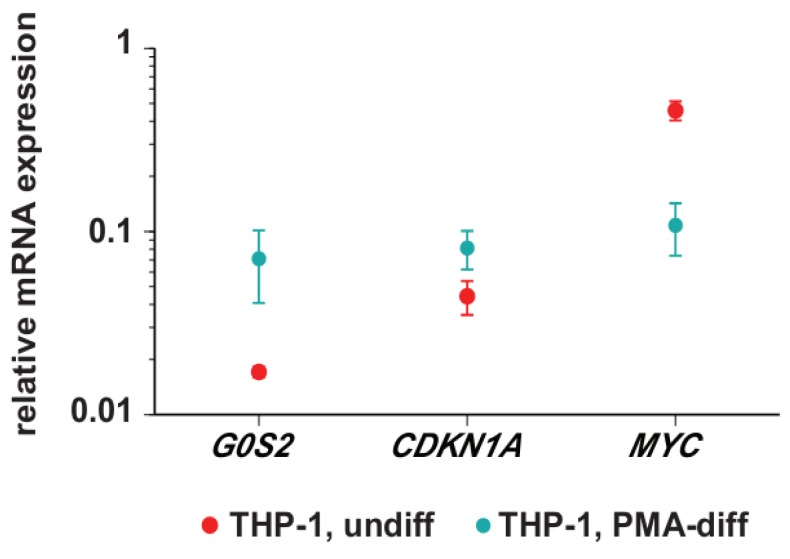
Basal mRNA expression of *G0S2*, *CDKN1A* and *MYC* in monocyte- and macrophage-like cells. qPCR was performed to determine the relative basal expression of the genes *G0S2*, *CDKN1A* and *MYC* (normalized to the reference genes *B2M*, *GAPDH* and *HPRT1*) in undifferentiated and PMA-differentiated THP-1 cells. The data points represent the means of three independent experiments and the bars indicate standard deviations.

In undifferentiated THP-1 cells the basal mRNA expression of the *G0S2* gene was 27-times lower than that of the *MYC* gene and 2.6-times reduced when compared with the *CDKN1A* gene (Supplementary Figure S1). In contrast, after THP-1 cells were differentiated by a 3-day treatment with PMA to M2-type macrophage-like cells, the expression of the three genes was very similar and differed by no more than 1.5-fold, *i.e.*, the differentiation process equalized the basal expression of the genes. This needs to be taken into account for the analysis of the following time course experiments.

**Figure 1:**
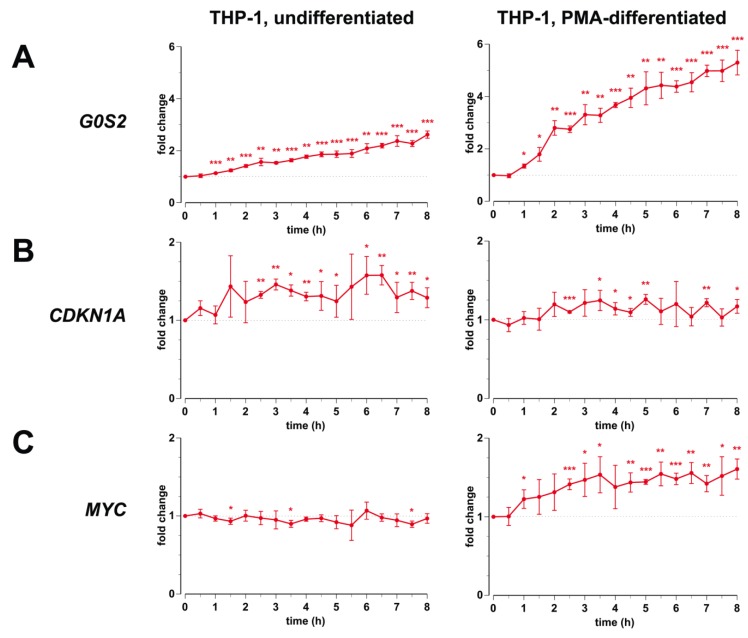
Primary response of *G0S2*, *CDKN1A* and *MYC* to 1,25(OH)_2_D_3_ in monocyte- and macrophage-like cells. qPCR was performed to determine the change of expression of *G0S2*, *CDKN1A* and *MYC* in response to incubation with 1,25(OH)_2_D_3_ over a time period of 8 h in undifferentiated THP-1 cells (left) and PMA-differentiated THP-1 cells (right). Data points represent the means of at least three independent experiments and the bars indicate standard deviations. Two-tailed Student’s t-tests were performed to determine the significance of the mRNA induction by 1,25(OH)_2_D_3_ (* *p* < 0.05; ** *p* < 0.01; *** *p* < 0.001).

We stimulated both undifferentiated and differentiated THP-1 cells with 1,25(OH)_2_D_3_ over a time course of 8 h (Figure 1) and observed that *G0S2* is both in monocyte- and macrophage-like cells a primary 1,25(OH)_2_D_3_ target gene, which is already significantly up-regulated after 1 h of ligand treatment (Figure 1A). Interestingly, despite a higher basal expression the *G0S2* gene is more inducible in PMA-differentiated THP-1 cells (5.3-fold after 8 h ligand treatment) than in undifferentiated THP-1 cells (2.6-fold). In both cell types also the *CDKN1A* gene was significantly induced by 1,25(OH)_2_D_3_, but reached during the 8-h time course only a maximal induction of 1.6-fold in monocyte-like cells and 1.3-fold in macrophage-like cells (Figure 1B). The *MYC* gene, which in most cellular systems is known to be a negatively regulated 1,25(OH)_2_D_3_ target gene [28], showed in monocyte-like cells during the 8-h time course no reasonable change in mRNA expression (Figure 1C). In contrast, in macrophage-like cells *MYC* turned out to be an early responding up-regulated (1.6-fold) 1,25(OH)_2_D_3_ target gene with a reduced basal expression level.

**Figure S2:**
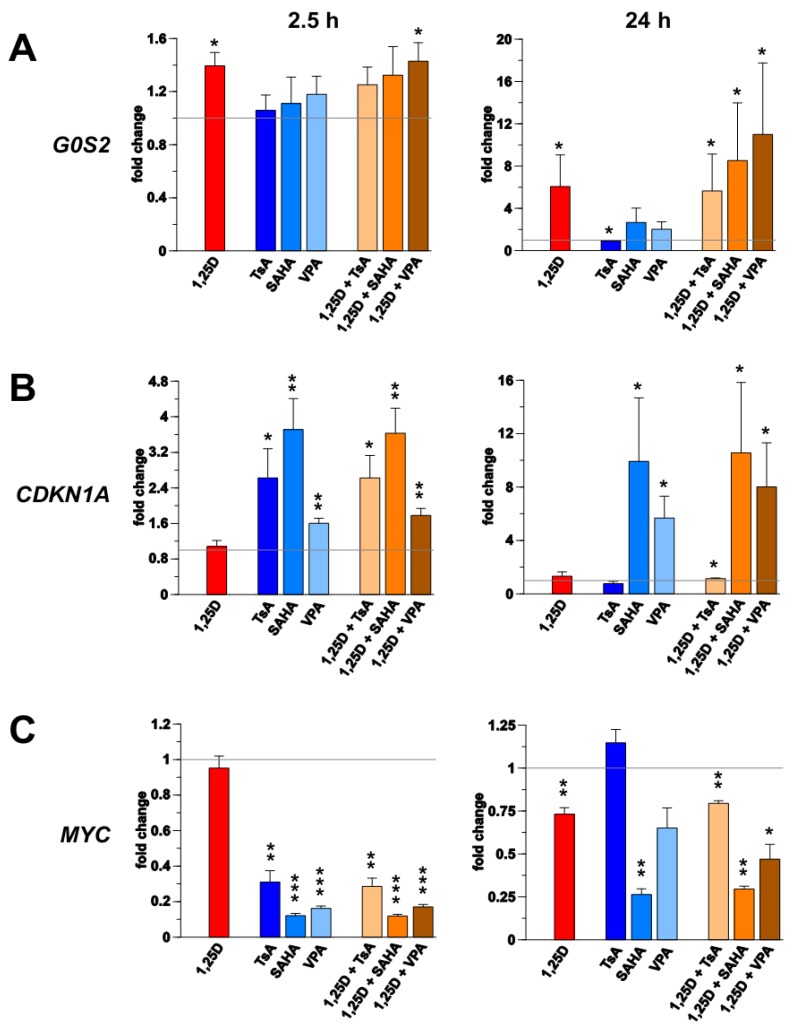
Short- and long-term effects of HDAC inhibition on *G0S2*, *CDKN1A* and *MYC* expression. qPCR was performed to determine the relative changes of mRNA expression of the genes *G0S2*, *CDKN1A* and *MYC* normalized by the three reference genes *B2M*, *GAPDH* and *HPRT1* in undifferentiated THP-1 cells in response to incubation with 1,25(OH)_2_D_3_ (1,25D), TsA, SAHA and VPA, alone or in combination, for 2.5 and 24 h. The columns represent the means of three independent experiments and the bars indicate standard deviations. Two-tailed Student’s t-tests were performed to determine the significance of the mRNA expression changes by the stimuli (* *p* < 0.05; ** *p* < 0.01; *** *p* < 0.001).

Reasoning that the differences in basal and regulated expression may reflect the epigenetic state of the gene loci, we characterized the transcriptional response of the three genes in the absence and presence of 1,25(OH)_2_D_3_ with the HDAC inhibitors TsA, SAHA and VPA after 2.5 and 24 h (Supplementary Figure S2). *G0S2* was confirmed to be an early responding, up-regulated 1,25(OH)_2_D_3_ target (1.4-fold after 2.5 h and 6.1-fold after 24 h) and was only faintly modulated by HDAC inhibitor treatment (Supplementary Figure S2A). In contrast, while also in this experimental series the response of the *CDKN1A* gene to 1,25(OH)_2_D_3_ stayed marginal, the gene was strongly up-regulated by HDAC inhibitors, in particular by SAHA, which induced the gene 3.7-fold after 2.5 h and 9.9-fold after 24 h (Supplementary Figure S2B). Inversely, the *MYC* gene was already after 2.5 h treatment strongly down-regulated by all three HDAC inhibitors (up to 8.3-fold), while after 24 h stimulation SAHA showed the most prominent effect (3.8-fold down-regulation). At this 24 h time point, 1,25(OH)_2_D_3_ treatment resulted in a statistically significant 1.4-fold down-regulation of the *MYC* gene (Supplementary Figure S2C).

In summary, the genes *G0S2*, *CDKN1A* and *MYC* respond to 1,25(OH)_2_D_3_ but with a differential profile: *G0S2* is more prominently up-regulated in macrophage- than in monocyte-like cells, while *CDKN1A* responds better in monocyte-like cells. Interestingly, *MYC* is weakly down-regulated in monocyte-like cells but up-regulated in macrophage-like cells. For comparison, in monocyte-like cells *G0S2* shows hardly any response to HDAC inhibitors, while *CDKN1A* is strongly up-regulated after a long-term treatment with the inhibitors and *MYC* is down-regulated already after a short-term treatment.

### 3.2. Genomic Profile of G0S2, CDKN1A and MYC in Monocyte- and Macrophage-like Cells

**Figure 2:**
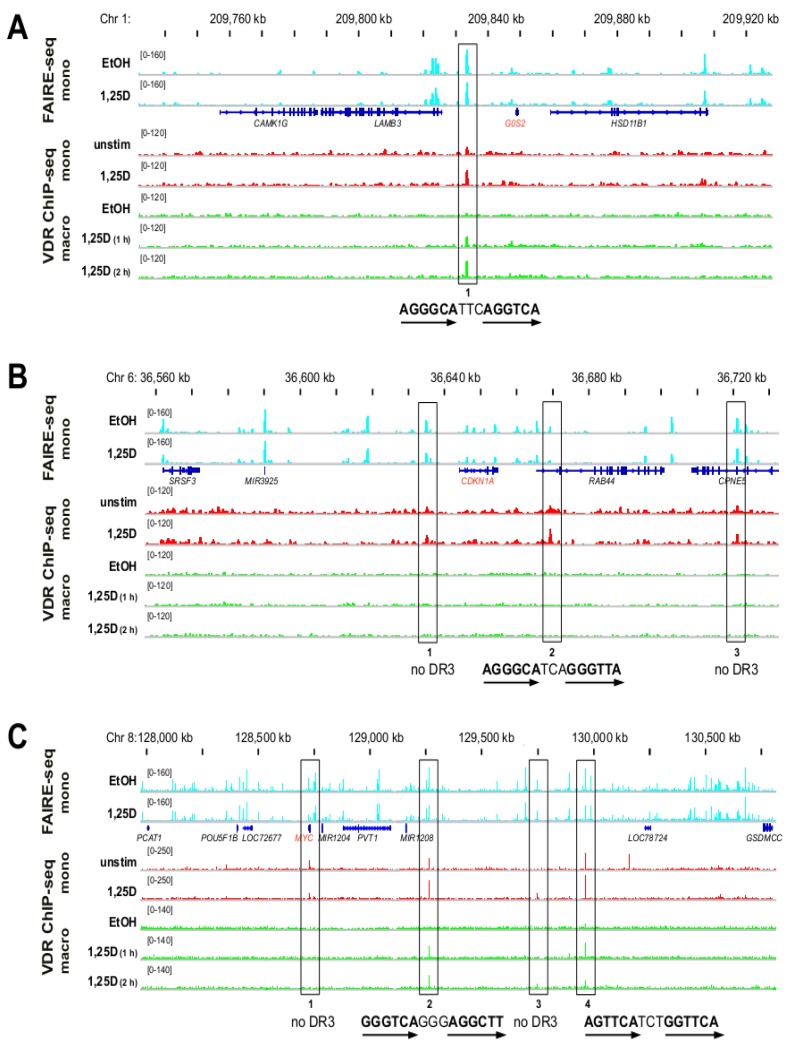
1,25(OH)_2_D_3_-dependent chromatin opening and VDR association in monocyte- and macrophage-like cells. The IGV browser was used to display the VDR peaks of the chromosomal domains (see Supplementary Figure S3) of the genes *G0S2.* (**A**) *CDKN1A* (**B**) and *MYC* (**C**). The peak tracks show FAIRE-seq data obtained from monocyte-like cells (mono, undifferentiated THP-1 cells, treated for 100 min with EtOH or 1,25(OH)_2_D_3_ (1,25D), light blue [34]) in comparison to VDR ChIP-seq data from monocyte-like cells (undifferentiated THP-1 cells, without or with 1,25(OH)_2_D_3_ treatment for 40 min, red [4]) and from macrophage-like cells (macro, PMA-differentiated THP-1 cells, treated with EtOH or 1,25(OH)_2_D_3_ for 1 and 2 h, green). The gene structures are shown in blue. Investigated VDR peak regions are boxed. The sequences of the DR3-type VDR binding sites below the summits of the peaks are indicated (arrows indicate the hexameric nuclear receptor binding sites); some peaks carry no DR3-type sequence.

**Figure 3:**
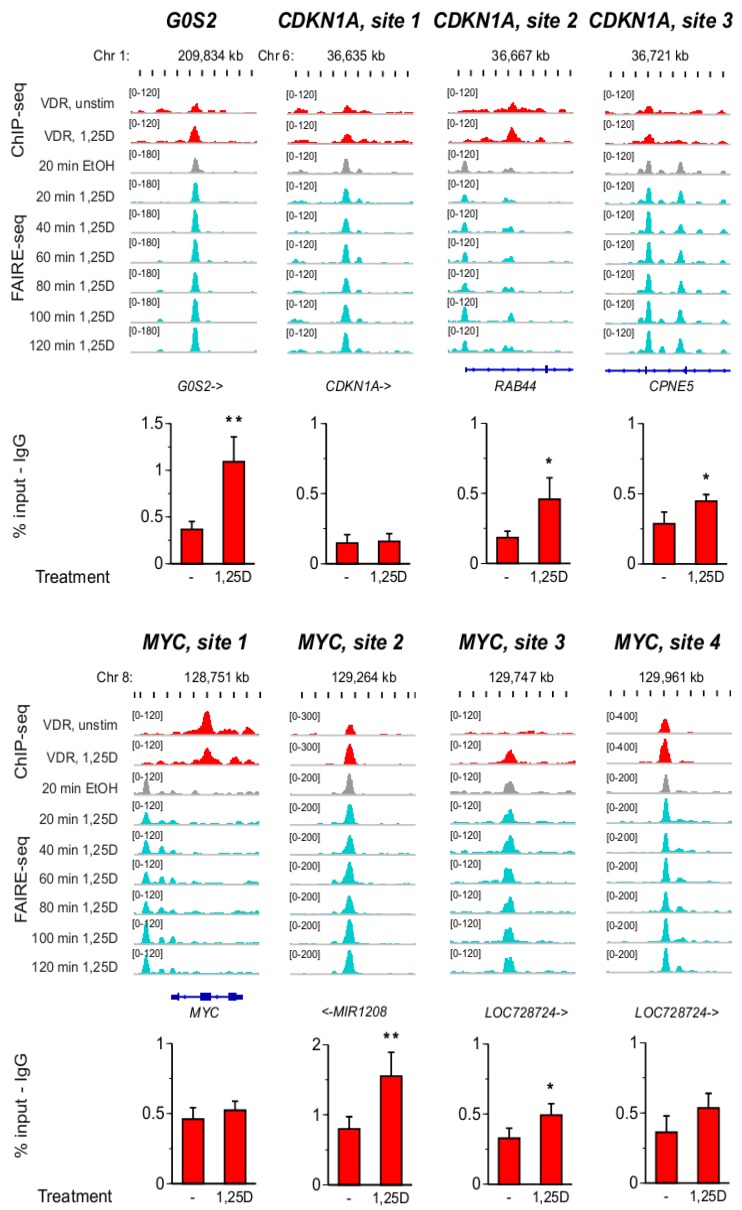
Detailed genomic view on VDR association and open chromatin. The IGV browser was used to visualize the loci of the genomic VDR binding sites (+/− 5 kb of the peak summit or, in case of site 3 of the *CDKN1A* gene, of the center of the two FAIRE peaks) of the genes *G0S2*, *CDKN1A* and *MYC*. The peak tracks display VDR ChIP-seq data from undifferentiated THP-1 cells (red, either unstimulated cells or treated for 40 min with 1,25(OH)_2_D_3_ (1,25D) [4]) and a time course of FAIRE-seq time course data from THP-1 cells (from cells treated with EtOH (grey) or 1,25(OH)_2_D_3_ (turquois) for the indicated time periods [34]). The gene structures are shown in blue. ChIP-qPCR was performed with chromatin samples obtained from undifferentiated THP-1 cells that were either unstimulated or treated for 2 h with 1,25(OH)_2_D_3_. Results show VDR association subtracted by unspecific IgG binding at all eight genomic regions. Columns represent the means of four independent experiments and the bars indicate standard deviations. Two-tailed Student’s t-tests were performed to determine significant 1,25(OH)_2_D_3_-induced VDR association in reference to untreated cells (* *p* < 0.05; ** *p* < 0.01).

**Figure S3:**
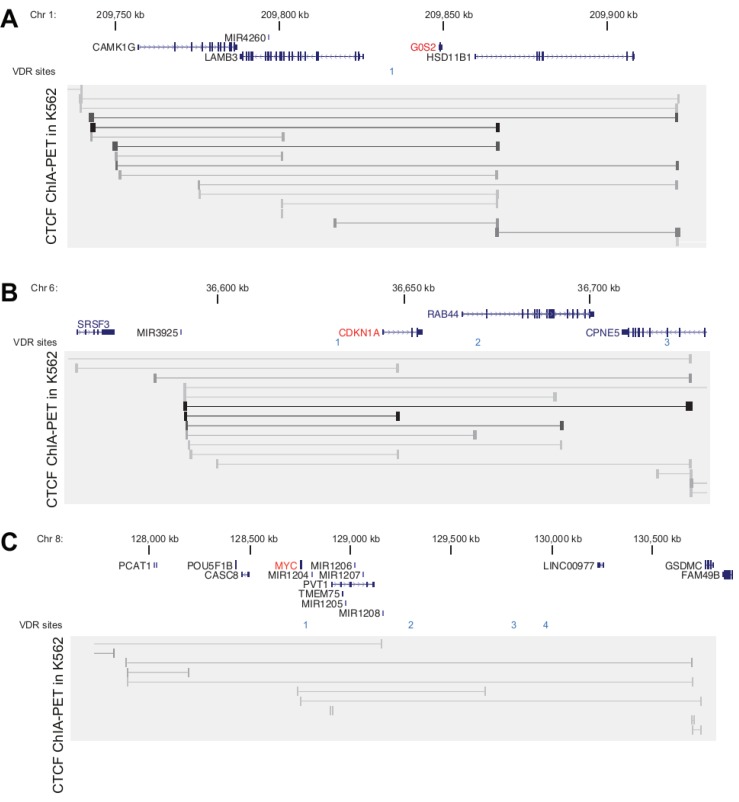
Looping between CTCF sites at the loci of *G0S2* (A), *CDKN1A* (B) and *MYC* (C). The UCSC genome browser (http://genome.ucsc.edu) was used to display CTCF ChIA-PET data from K562 cells [32] for the same genomic region as shown in Figure 2. Horizontal lines represent chromosomal domains formed by DNA looping between CTCF sites. The positions of the VDR peaks are indicated by numbers and the gene structures are shown in blue.

The VDR ChIP-seq dataset from undifferentiated THP-1 cells [4] contains several peaks in reasonable vicinity to the TSS regions of the genes *G0S2*, *CDKN1A* and *MYC*. In order to determine, which of these VDR binding site candidates may be involved in the regulation of the genes, we first estimated the size of the chromosomal domains containing the respective TSS regions. Borders of chromosomal domains are defined by DNA looping mediated by the transcription factor CCCTC-binding factor (CTCF) [29]. CTCF binding sites are often highly conserved between tissues and cell lines [30]. The human monocytic leukemia cell line K562 is reasonably similar to THP-1 cells [31]. Importantly, for this cellular model a genome-wide map of the 3-dimensional interactions of CTCF is available, which was determined by chromatin interaction analysis with paired-end tag sequencing (ChIA-PET) assays [32]. We used the UCSC genome browser to display the respective CTCF looping data for each of the three genes (Supplementary Figure S3). For the *G0S2* locus on chromosome 1 the largest loop spans over 170 kb and contains one VDR binding site (Supplementary Figure S3A, for more details see Figure 2A and Figure 3). The respective chromosomal domain for the *CDKN1A* gene on chromosome 6 seems to cover only 140 kb (in maximum 170 kb, see Supplementary Figure S3B) and contains three VDR binding sites (see also Figure 2B and Figure 3). In contrast, the chromosomal domain around the *MYC* gene on chromosome 8 appears to be as large as 2.3 Mb and contains four VDR binding sites (Supplementary Figure S3C, more details in Figure 2C and Figure 3). 

Next, we used the IGV browser and displayed for the chromosomal domains (see Figure S3) open chromatin in undifferentiated THP-1 cells, as determined by FAIRE-seq [33,34], and VDR ChIP-seq data from undifferentiated THP-1 cells [4] and from PMA-differentiated THP-1 cells (Figure 2). The single VDR binding site of the *G0S2* gene, which is located 15 kb upstream of the gene’s TSS in a region of open chromatin, was found both in monocyte- and macrophage-like cells and carries a DR3-type sequence within the summit region of the peak (Figure 2A). All three VDR binding sites of the *CDKN1A* gene, which are located 9 kb upstream and 25 and 77 kb downstream of the gene’s TSS in regions of open chromatin, were observed only in monocyte- and not in macrophage-like cells (Figure 2B). Only site 2 of the *CDKN1A* gene contains a DR3-type sequence. All four VDR peaks around the *MYC* gene, which are located 2.7, 514, 1,000 and 1,210 kb downstream of the gene’s TSS, were observed in monocyte-like cells (Figure 2C). Sites 2, 3 and 4 of the *MYC* gene but not site 1 close to the TSS were associated with open chromatin in monocyte-like cells, while only sites 2 and 4 were bound by VDR in macrophage-like cells. Only the two latter sites carry each a DR3-type sequence.

Taken together, the chromosomal domains of the genes *G0S2* and *CDKN1A* are with 140–170 kb rather compact and contain one or three VDR binding sites, respectively. In contrast, the chromosomal domain of the *MYC* gene is at least 15 times larger and accommodates four VDR binding sites. All eight VDR binding sites are found in monocyte-like cells, seven of them are also associated in the same cellular model with open chromatin. Four of these seven VDR binding sites carry a DR3-type sequence, three of which are also occupied with VDR in macrophage-like cells.

### 3.3. VDR Binding Sites of G0S2, CDKN1A and MYC

For a detailed view on the VDR binding sites of the genes *G0S2*, *CDKN1A* and *MYC* we displayed VDR ChIP-seq data from undifferentiated THP-1 cells [4] in comparison to FAIRE-seq time course data from the same cellular model [34] (Figure 3). 

**Figure S4:**
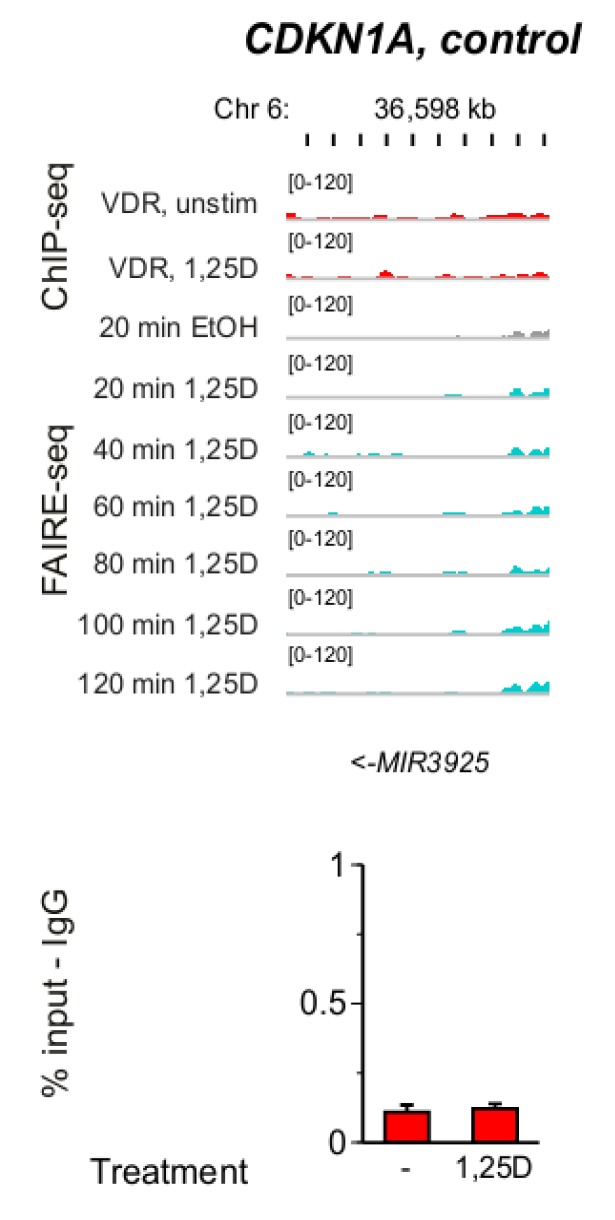
ChIP-qPCR control region. Same setup as in Figure 3 for the control region of the *CDKN1A* locus was applied.

Interestingly, site 1 of the *MYC* gene shows the unique property that VDR binding reduced after stimulation with 1,25(OH)_2_D_3_, while in parallel the region is not associated with open chromatin. At the seven other regions ligand-inducible VDR binding and association with open chromatin was found. However, only at the VDR binding site of the *G0S2* gene 1,25(OH)_2_D_3_-dependent chromatin opening was observed (compare the turquois with the grey tracks in Figure 3). In parallel, we performed ChIP-qPCR with chromatin templates from undifferentiated THP-1 cells, which were either unstimulated or had been treated for 2 h with 1,25(OH)_2_D_3_. In reference to a negative control region located 46 kb upstream of the *CDKN1A* gene (Supplementary Figure S4), seven of the investigated regions showed statistically significant association with VDR, while site 1 of the *CDKN1A* gene was very weak. Significant ligand-induced increase of VDR binding was found only for the VDR site of the *G0S2* gene, for sites 2 and 3 of the *CDKN1A* gene and for sites 2 and 3 of the *MYC* gene. However, the ligand-induced reduction in VDR binding at site 1 of the *MYC* gene as well as the strong, ligand-inducible VDR binding to site 4, which had been observed by ChIP-seq, could not be reproduced by ChIP-qPCR.

In summary, specific VDR association could be observed at all eight investigated regions, independently of whether they contained a DR3-type sequence or not. Also the enrichment of VDR was in certain cases sensitive to the cell status (monocyte- or macrophage-like cells). At site 1 of the *MYC* gene ChIP-seq and FAIRE-seq data suggest a different profile than at the seven other sites.

## 4. Discussion

The availability of genome-wide data, obtained by individual research teams or by larger consortia, such as ENCODE [35], provide a new view on the regulation of genes. In this study, we combined insight gained from microarray and ChIP-seq datasets obtained in the leukemia cell line THP-1 [4]. We selected the genes *G0S2*, *CDKN1A* and *MYC* as master examples for demonstrating the complex role of 1,25(OH)_2_D_3_ in the control of cellular proliferation in this cancer cell model. In this context, we exhibited the utility of publically available data from ENCODE, in order to limit the size of the genomic regions that needed to be screened for VDR binding sites.

The oncogene *MYC* was one of the first genes to be reported as an 1,25(OH)_2_D_3_ target [36]. The Myc oncoprotein is a critical regulator of cell cycle progression (reviewed in [37]) but also controls the induction of apoptosis [38]. Depending on the cellular model the Myc protein acts either as a pro- or an anti-survival factor [39]. Most studies on the effects of 1,25(OH)_2_D_3_ on *MYC* gene expression report its down-regulation. This suggests that 1,25(OH)_2_D_3_, via the down-regulation of *MYC* gene expression, may lead to the inhibition of cellular growth and induction of apoptosis. This study agrees with most other studies that the effects of 1,25(OH)_2_D_3_ on the down-regulation of *MYC* mRNA expression are rather modest. However, given the importance of this protein, even modest changes in expression could yield significant biological effects. Therefore, the well-documented effects of 1,25(OH)_2_D_3_ and its synthetic analogues on the inhibition of cellular proliferation (reviewed in [40]) and the induction of apoptosis [41] probably reflect a combination of modest *MYC* down-regulation with increased expression of proteins that exert mitotic restraint. The tumor suppressor gene *CDKN1A* encodes for the well-known cell cycle inhibitor protein p21^WAF1^ (reviewed in [42]) and has been implicated for at least 20 years as a 1,25(OH)_2_D_3_ target gene [43,44]. For *CDKN1A* most studies agree on an up-regulation of the gene by 1,25(OH)_2_D_3_, which should then result in cell cycle arrest and the induction of differentiation [43]. However, similar to the *MYC* gene, the reported effects of 1,25(OH)_2_D_3_ on *CDKN1A* up-regulation are rather modest and will also not explain most of the cell regulatory properties of VDR ligands.

The pre-genomic approaches of VDR target gene candidates described above provide only a limited understanding of the anti-proliferative actions of VDR ligands. Nevertheless, it is interesting and important that also genomic and transcriptomic studies list *MYC* and *CDKN1A* as VDR target genes, even though along with hundreds of other genes. The THP-1 leukemia model revealed at least 408 statistically up-regulated genes and 230 down-regulated genes after 4 h of stimulation with 1,25(OH)_2_D_3_ [4]. Most of these genes have no obvious relation to cell cycle regulation but, based on Gene Ontology terms, rather relate to immune or metabolic function [4]. This would suggest that, at least in the THP-1 leukemia model, the primary effects of 1,25(OH)_2_D_3_ are rather in the area of immune function and metabolism than in cell cycle control. This may depend on the cell type, though.

Many recent studies have shown that apparent distinct physiological actions, such as immune function, metabolism and cellular growth, are closer interlinked than assumed before. This means that many genes have a dual or even triple function in physiology. *G0S2* is such an example of a gene being not exclusively associated with one physiological function. Reflected by its name, the gene was initially associated with the re-entry of cells from the G_0_ phase to the G_1_ phase, *i.e.*, with a clear cell cycle regulatory function, but nowadays the G0S2 protein appears to be multifaceted being involved in proliferation, apoptosis and carcinogenesis but also in inflammation and metabolism [45]. Interestingly *G0S2* is reported to be a target gene for the nuclear receptors peroxisome proliferator-activated receptors [46] and retinoid acid receptors [47], which supports a concept of combinatorial actions of nuclear receptors.

In this study, mRNA inductions showed that the *G0S2* gene was clearly more responsive to 1,25(OH)_2_D_3_ treatment than the genes *CDKN1A* and *MYC*. Contrary to many observations that indicate a higher responsiveness of a gene when it is relatively low expressed, the 1,25(OH)_2_D_3_ inducibility of the *G0S2* gene even increased after its basal expression raised during the differentiation of monocytic THP-1 cells into macrophage-like cells. This suggests that in macrophage-like cells the *G0S2* gene may be more extensively involved in its non-cell cycle-related functions than in monocyte-like cells. The prominent binding of VDR to the single binding site of the *G0S2* gene locus in macrophage-like cells supports this view. The unexpected induction of the *MYC* gene in macrophage-like cells by 1,25(OH)_2_D_3_ may suggest that the Myc protein has a different function in non-proliferating macrophages than in proliferating monocytes. In parallel, in macrophage-like cells VDR appears to bind only to two of its four binding sites within the *MYC* gene locus. Importantly, site 1, which is close to the *MYC*’s TSS and is not associated with open chromatin, is not used in macrophage-like cells. This suggests the intriguing possibility that this VDR binding region, being located close to the TSS, plays a major role in the down-regulation of the *MYC* gene. Finally, the basal expression of the *CDKN1A* gene increased during the differentiation of monocytes into macrophage-like cells and in parallel the responsiveness of the gene to 1,25(OH)_2_D_3_ was reduced. This fits with the observation that the macrophage-like cells are not proliferating anymore and that in them VDR occupies none of its three binding sites within the *CDKN1A* gene locus.

In the past, the limited amount of sequence information and genome annotation led to the assumptions that (I) a transcription factor should bind to the promoter region upstream of the gene’s TSS to regulate mRNA expression and (II) even enhancer regions are in rather close vicinity to the TSS. At latest the results of the ENCODE project [35] have significantly changed this view. A transcription factor, such as VDR, will equally likely bind upstream and downstream of the TSS and the binding site can be many hundred kb in distance. This raised the question, whether there is any limit in the distance, from which a transcription factor will activate a gene. We suggest that the use of ChIA-PET data derived from CTCF and other insulator-like proteins may allow a good estimation of the size of a gene’s true chromosomal domain, encompassing the transcription factor binding sites and the TSS region. In proof of principal studies we took advantage of the publically available CTCF ChIA-PET dataset from K562 cells, which is one of the main cellular models of the ENCODE project [35]. Although K562 cells are more erythroid-like, they are closest to THP-1 cells amongst the more than 100 cell lines used in the ENCODE project. Moreover, in contrast to most other transcription factors, the binding of CTCF is conserved throughout many tissues and cell types. The sizes of the chromosomal domains of the *G0S2* and *CDKN1A* gene loci are in the same order (140–170 kb), while that of the *MYC* gene is far larger. The latter may be related to the fact that the gene density of the *MYC* locus is significantly lower than that of most other genes. This feature gives at this locus a lot of space for a number of long-ranging chromatin loops to be formed between rather distant regions, such as the reported regulation of *MYC* by a very distant binding site of the transcription factor TCF4 [48].

Due to their more generic function in the 3-dimensional organization of chromatin, CTCF binding sites may represent an extreme in the conservation between tissues and cell types. In contrast, most other transcription factors, such as VDR, show a far more tissue-specific genomic binding pattern. As demonstrated in this study at the example the two rather closely related cellular models, undifferentiated THP-1 cells and PMA-differentiated THP-1 cells, only three or eight VDR binding sites are occupied in each of the two cell types. Therefore, it is no surprise that for the same gene different patterns of VDR binding sites are detected, when shifting from one cellular system to another. For example, ChIP-seq in LS180 colon cancer cells indicated VDR binding sites 139, 146 and 335 kb upstream of the *MYC* TSS [5], while ChIP-qPCR in RWPE-1 normal human prostate cells suggested VDR binding 1.3 kb upstream of the gene’s TSS and at site 1 (2.7 kb downstream of the TSS). Similarly, in MCF7 human breast cancer cells and in RWPE-1 cells ChIP-qPCR suggested VDR binding to the *CDKN1A* locus TSS, and to sites 2.2 and 4.7 kb upstream of it [49,50]. An additional divergence between primary 1,25(OH)_2_D_3_ target genes is the different number of VDR binding sites used in their regulation. The *G0S2* gene is a simple example with only one VDR binding site within its chromosomal domain, which is used in all published VDR ChIP-seq data sets besides that from colon cancer cells. In contrast, the *CDKN1A* gene uses in THP-1 cells three VDR binding sites, of which no. 1 and 3 are also occupied in lymphoblastoid cells [3], and the *MYC* gene even four VDR binding sites, none of which are used outside the monocyte/macrophage cellular model. This limited set of examples suggests that—with increased dissection of experimental models representing different tissues and disease states—for VDR binding there is increasingly observed tissue-specific complexity.

## 5. Conclusions

The increasing number of 1,25(OH)_2_D_3_ target genes being involved in the control of cellular growth makes it obvious that no single gene has the potential to play a dominant role in this process, *i.e.*, there will be no chance for a single gene-targeted therapy. The example of three primary VDR target genes, *G0S2*, *CDKN1A* and *MYC,* with important function in cellular growth suggests that each gene has an individual and cell-specific scenario in the regulation by 1,25(OH)_2_D_3_ and its receptor VDR. However, there are also unifying principles, such as the dependence or independence from DR3-type VDR binding sites, which may allow a refinement of therapies.

## Supplementary Materials

**Table S1:** Reverse transcription qPCR primers.

Gene	Fragment size (bp)	Annealing temperature (°C)	Primer sequences (5'-3')
*B2M* ^1,2^	246	60	GGCTATCCAGCGTACTCCAAA CGGCAGGCATACTCATCTTTTT
*CDKN1A* ^3^	99	60	GCCACTAAGGTCATTCCCGCCT CCTTGCGCTTCTGGGCCATCAT
*G0S2* ^4^	102	60	GCCACTAAGGTCATTCCCGCCT CCTTGCGCTTCTGGGCCATCAT
*GAPDH* ^1,2^	113	60	CATGAGAAGTATGACAACAGCCTAGTC CTTCCACGATACCAAAGT
*HPRTI* ^1,5^	94	60	TGACACTGGCAAAACAATGCA GGTCCTTTTCACCAGCAAGCT
*MYC* *^3^*	147	60	CCAGCAGCGACTCTGAGG GGACCAGTGGGCTGTGAG

^1^ reference gene; ^2^ Sequence obtained from PrimerBank (http://pga.mgh.harvard.edu/primerbank); ^3^ Designed with Oligo 4.0 software (National Biosciences, Plymouth, MN, USA); ^4^ Designed using Primer-BLAST (www.ncbi.nlm.nih.gov); ^5^ see.

**Table S2:** ChIP-qPCR primers.

Genomic region	Fragment size (bp)	Annealing temperature (°C)	Primer sequences (5'-3')
*G0S2* ^1^	119	62	GCCTGAGAGTATGCTGTGTACGTTT CTAAGTGCTCTCTTGGCGTAACAAA
*CDKN1A* ^2^, peak 1	129	58	TGTGGGGAGGGTGTTTCAG GAGGGAAGGAAGGAGTGAG
*CDKN1A* ^2^, peak 2	91	58	CAGAGGAAGTGGGTTGAG AGCAGGGCAGGAGAGATTATAC
*CDKN1A* ^2^, peak 3	69	62	GCACTCTTGACCTTGACGGA CTAACACCCTTGGCTTGGAC
*CDKN1A* ^2^, control	180	62	ATCACAGGGGTCAGCACATC CGCAGCATTTGGGTTCACAC
*MYC* ^2^, peak 1	181	62	GTCACACCCTTCTCCCTTC CGCTCCACATACAGTCCTG
*MYC* ^2^, peak 2	93	58	CCACACTAACCTCTCAGTTC GTAATGATACTCCCAGCAAAG
*MYC* ^2^, peak 3	137	62	GGATGTCAGCAGGGTTTCTC GGAAGTGATTTCGGGAGTAG
*MYC* ^2^, peak 4	96	58	GCTCTGTTGGTGTGGACTG GATTAGGGTGCCATAGAATAC

^1^ Designed using Primer-BLAST (www.ncbi.nlm.nih.gov); ^2^ Designed with Oligo 4.0 software.
